# Capacity building through cross-sector partnerships: a multiple case study of a sport program in disadvantaged communities in Belgium

**DOI:** 10.1186/s12889-015-2605-5

**Published:** 2015-12-29

**Authors:** Mathieu Marlier, Steffie Lucidarme, Greet Cardon, Ilse De Bourdeaudhuij, Kathy Babiak, Annick Willem

**Affiliations:** Department of Movement and Sports Sciences, Ghent University, Watersportlaan 2, 9000 Ghent, Belgium; School of Kinesiology, University of Michigan, 1402 Washington Heights, 48109 Ann Arbor, MI USA

**Keywords:** Capacity building, Cross-sector partnerships, Disadvantaged communities, Community sport

## Abstract

**Background:**

Recent research has illustrated the need for cross-sector partnerships to tackle multidimensional problems such as health inequalities and sport and physical activity promotion. Capacity building is based on partnerships and has demonstrated effectiveness in tackling these multidimensional problems. This study aims to explain how cross-sector partnerships build capacity at the practitioner, organisational and partnership levels. The subject of this study is a community sport program (CSP) that aims to increase sport participation rates and physical activity levels.

**Methods:**

The study examined multiple cases in four disadvantaged communities in Antwerp, Belgium where the CSP was implemented. Forty-four face-to-face interviews were held with leaders from sport, social, health, culture and youth organisations that collaborated with the CSP.

**Results:**

Thirteen elements of cross-sector partnerships were identified as critical to building capacity at each of the different levels. These include: process evaluation, trust, mutuality, policy support, partner complementarity and fit, diversity of activities and period of collaboration-time. Trust in turn was fostered by a longer period of collaboration-time, better personal contact, clearer coordination and an external focus. Policy support was developed by support of partners and establishing clear metrics of success.

**Conclusion:**

Insight into the key elements of cross-sector partnerships that build capacity is given and several practical recommendations are suggested for practitioners and policy makers.

## Background

Health and illness follow a social gradient: the lower the socioeconomic position, the less people are healthy [1]. Tackling these health inequalities is a major concern to most public health organisations and governments [2]. A key challenge in dealing with these inequalities exists in acting on the social determinants of health. In recent years, focus has shifted from interventions at the individual level to interventions at the community level in order to improve the social determinants of health [3]. These interventions need to be strengthened by community insight and the mobilization of resources to solve locally identified health problems [1].

Sport has emerged as a potential strategy to capture or ‘hook’ the interest of a large group of people, even in disadvantaged communities [4–6]. Participation in sport has furthermore been associated with higher levels of physical activity, better mental health [7], and higher social capital [8]. In light of these findings, health, social and other organisations have shown a growing interest in using sport or collaborating with organisations in the sport sector to increase physical activity, enhance mental health or engage civic participation in their communities [9]. Moreover, it is generally acknowledged that partnerships among a wide range of organisations are required to deal with multidimensional problems and challenges, such as sport and physical activity promotion [10] and addressing health inequalities [11].

One approach that makes use of cross-sector partnerships and has demonstrated effectiveness in tackling health inequalities in physical activity and sport participation is capacity building [12, 13]. Capacity building has been defined in the WHO health promotion glossary as “the development of knowledge, skills, commitment, structures, systems and leadership to enable effective health promotion.” [14] (p 341) It influences three levels of health promotion. First, it affects the practitioner level by enhancing their individual knowledge and skills. Second, it stimulates the organisational level by expanding support and infrastructure. Third, it impacts the partnership level by building and/or strengthening partnerships and cohesiveness among the health promotion organisations [14]. On a side note, in the management literature, capacity generally refers to organizational capacity. It is important to stress that capacity in this study, following the definition of the WHO [14], refers not only to organizational capacity but also to capacity of practitioners and capacity of the partnership.

Although the importance of partnerships in capacity building programs to promote physical activity and sport have repeatedly been emphasized [15–18], no studies have focused on the specifics of how these partnerships build capacity at the practitioner, organisational and partnership levels. The present study attempts to fill this gap by identifying the key elements of cross-sector partnerships that build capacity at these levels. To reach this aim the present study investigated a community sport program (CSP) that makes use of cross-sector partnerships to build capacity.

A prior study showed that this CSP was related to higher levels of sport participation [19]. In communities where the CSP was implemented, 61.3 % of adults engaged in sport, whereas in similar communities, without the CSP, this was only 42.4 %. In the present study, sport participation was defined as ‘physical activities that require a sufficient rate of exertion and that take place in an athletic context during leisure time’ [20] (p. 143). It referred both to organized as well as non-organized and individual as well as team sport activities. In general older adults, women from ethnic minorities and people from lower social classes were found to participate less in sport. However all of these groups reported higher sport participation rates in CSP communities [19]. Overall, the large majority indicated to sport on a recreational level (91.4 %).

This study will thus focus on which elements were most crucial in cross-sector partnerships to build capacity at the practitioners, organisational and partnership levels in the context of this CSP.

## Methods

### Description of the Community Sports Program (CSP)

The focus of the study was a community sport program (CSP) in Antwerp, Belgium (506,225 inhabitants). In the current study community refers to a specific geographical area. This CSP was established through a bottom-up process of trial and error by sports, social, youth and health care practitioners. It developed organically over the last 20 years by responding to local needs. Since 2003 the CSP has been managed and implemented by the Antwerp Sports Administration with the objective to increase sport participation rates for people in disadvantaged communities who experience higher financial, mobility and commitment thresholds to engage in sports. At the moment a total of 33 full-time equivalent (FTE) staff members are employed to deliver the CSP in Antwerp.

The Antwerp Sports Administration has five main tasks in delivering the CSP. These include: (a) receiving and giving information from and to the different sports, social, health, cultural and youth partners in the community; (b) supporting the sport activities of partners; (c) organizing sport activities complementary to those already offered by the partners; (d) creating new sport infrastructure in the community; (e) searching for new ways to reach their goals by being innovative. Currently, 17 communities (of the existing 62 communities in Antwerp) are implementing the CSP. Three coordinators manage the CSP at the city level. They coach and guide 30 staff members delivering the CSP in the 17 communities and they collaborate with the leaders of partner organisations in the areas of sport, social, health, cultural and youth development.

### Research design

The multiple case design used in this study made it possible to compare and unravel the key elements of cross-sector partnerships that build capacity at the practitioner, organisational and partnership levels in the different communities. This approach also provides a stronger case for theory building [21]. Furthermore, it enabled us to account for three frequently mentioned limitations that hamper progress in defining the key elements of cross-sector partnerships.

First, the stage of development of the program has generally not been considered in empirical research [22]. This study therefore investigated multiple cases: two Program Communities (PC 1 and PC 2) where the CSP had been implemented since 1998, and two (PC 3 and PC 4) where the program started in 2007.

Second, opinions of multiple stakeholders at different administrative and implementation levels of the program are frequently not taken into account [23]. This limitation was accounted for by collecting qualitative data of community sport, health, social, culture and youth partners both at the community and city levels. Examples of these partners are provided in Table 1.

**Table 1:** Examples of stakeholders in the different sectors at community and at city level

Stakeholders	Community level	City level
Sport	Sport clubs, local sport administrations, sport facility administration (e.g., swimming pools)	Department of sport events, the department of sport club support, department of school sport support
Health	Local health centres	/
Social	Outreach organizations, organizations fighting against drug abuse and homelessness, organizations focussing on building community cohesion and empowering disadvantaged individuals	Organization in charge of integrating new residents, the organization in charge of welfare affairs, the umbrella governing body of all community organisations dealing with people in poverty
Cultural	Organizations focussing on cultural activities (e.g., concerts, art workshops), organizations creating places to meet for community members	Governing body of social and cultural affairs
Youth	Outreach organizations for youth, organisations focusing on providing leisure opportunities for children, day-care organizations, juvenile delinquency prevention organizations	/

Finally, empirical evidence of the outcomes of partnerships at the population level is often lacking [24]. To account for this critique a component of our broader project included a study that explored the question of whether communities with a CSP had higher levels of sport participation than control communities without a CSP. The results of this study showed that program communities noted an average sport participation rate of 61.3 %, which was about 20 % higher than the control communities [19]. This present study tries to pinpoint the reasons and the underpinning processes of partnerships that build capacity on the three different levels and consequently help in explaining these proximal outcomes of the CSP.

### Data collection

Qualitative data were collected through in-depth, semi-structured interviews at the community (geographical area) and city levels. Sampling of participants was done by asking the CSP staff members which organizations and which individuals in these organizations they considered to be their most important partners in the community and in the city. At the community level the representatives of the organizations involved the practitioners who carry out the tasks set by the organizations on the field. Interview questions were built from a literature review [25] based on the framework of Parent and Harvey [26]. This framework has proven useful in identifying key elements of physical activity promotion through community partnerships [25]. It encompasses variables that have proven their relevance in previous research including [25]: (a) Antecedents (variables concerning the formation of the partnership); (b) Management (variables that relate to the functioning of the partnership); (c) Evaluation (variables that relate to the evaluation of the program and the partnerships). In total 44 interviews were conducted with community sport (CSP), sport (SP), social (SO), culture (CU), health (HE) and youth (YO) partners. At the community level 33 partners were interviewed in four different program communities, at the city level 11 partners were interviewed. Member checking was executed in two ways. First, by restating or summarizing answers of interviewees in case the researchers were not clear on interpretation of the response. Second, by communicating the preliminary analysis to all the participants in order to verify and confirm the preliminary findings of the analysis [27]. Interviews lasted on average 40 minutes. Informed consent was obtained for al interviewees. The study was approved by the Ethics Committee of the Ghent University Hospital. Table 2 presents an overview of the different partners for the selected communities and the city. It should be noted that partner organizations varied over the different communities according to the availability of suitable partners.

**Table 2:** Overview of organisations of study participants (interviewees)

	PC 1	PC2	PC3	PC4	City	Total
Members of CSP (CSP)	2	2	2	2	3	11
Sport Organisation (SP)	1	1	3	2	3	10
Social 0rganization (SO)	2	3	3	1	4	13
Cultural organisation (CU)	2	/	/	1	1	4
Health Organisation (HE)	1	1	/	/	/	2
Youth Organisation (YO)	1	3	/	/	/	4
Total	9	10	8	6	11	44

*CSP* Community Sport Program, *PC* Program Community

### Analyses

Qualitative data were analysed with Nvivo 10. Four steps were taken to reduce and analyse the 231,470 words of interview transcripts. First, a codebook was developed, based on the variables expressed in Parent and Harvey’s framework [26].

Second, text fragments were coded to the rightful nodes of the codebook. To assure quality of this coding process, another experienced researcher assisted, in case of doubt, in assigning certain text fragments to the proper node [28]. When new elements recurred in several interviews new nodes were inductively added. An example of a new node is external focus – namely, reaching own organisational goals by helping in the activities of partners. Combining both a deductive and inductive coding approach enabled the researchers to use the richness of previous literature and theory and extend this theory with new elements derived from the raw data [29]. Inter-rater reliability measured by kappa-coefficient, was 0.75. This coefficient represents the reliability between coding of the main researcher and the coding of a sample of interviews of a second researcher. Although guidelines are arbitrary, a kappa-coefficient of 0.75 is generally accepted as a good inter-rater reliability score [30].

In the third and crucial step of the analysis we looked for patterns in the variables of cross-sector partnerships and how they built capacity on the practitioner, organisational and partnership levels. Thus, more specifically, we looked for patterns in the coded variables and how they enhanced knowledge and skills among practitioners, how they expanded support and infrastructure to the organisations and how they built and strengthened partnerships and cohesiveness among the different organisations.

Finally, the most recurring and important patterns were used to identify the key elements of cross-sector partnerships to build capacity.

## Results

In total 13 key elements of cross-sector partnerships were identified that build capacity at the different levels. Table 3 summarizes these different key elements per level. Eight key elements of partnership capacity building were deductively derived based on the work of Parent and Harvey [26]: process evaluation, trust, coordination, mutuality, partner complementarity and fit, personal contact, period of collaboration time and policy support. Four key elements deductively emerged from the analysis: external focus, metrics for success, support of partners and diversity of activities. The next section describes each key element of the cross-sector partnerships and includes representative quotes to illustrate how capacity was built at the practitioner, organisational and partnership levels.

**Table 3:** Key elements of cross-sector partnerships that build capacity at the practitioner, organisational, and partnership levels

Capacity Building Level	Key elements of cross-sector partnerships	Explanation of how capacity is build by the key element at the given level
Practitioner	Process evaluation	Assessment of activities during and at the end of the project to see where improvements can be made.
	Trust	Confidence in abilities and intentions of partners. Higher trust leads to more knowledge and skill sharing.
	Period of collaboration-time	Duration of partnerships. Trust needs time to be developed. In a good partnership more skills and knowledge will be shared as time goes by and trust increases.
	Personal contact	Personal relationship between people of different organisations. Open attitude and commitment to the partnership improve the personal contact, trust and knowledge sharing.
	Coordination	Clarity of role, task, and expected input from partners increases accountability, trust and knowledge sharing among partners
	External focus	Reaching own organisations goals by engaging in activities of other partners multiplies trust and knowledge sharing
Organisational	Mutuality	Interdependence between the partners. Greater needs to collaborate leads to greater willingness to share resources.
	Policy support	Extent to which policy supports the organisation and allocates financial resources.
	Support of partners	Partners who indicate added value of the partnership create legitimacy and positively influence policy makers.
	Metrics for success	Objective results of relationships create legitimacy and positively influence the policy makers.
Partnership	Partner complementarity and fit	Composition of network partners with different expertise, so complementary skills and knowledge can be shared.
	Diversity of activities	Multiple activities create added value for a wide variety of partners and extends the network
	Period of collaboration-time	Duration of partnership gives time to obtain results and convince potential partners of the added value of a relationship.

### Key elements of cross-sector partnerships that build capacity at the practitioner level

Two key elements of cross-sector partnerships were identified to build capacity at the practitioner level: process evaluation and trust. These elements were found crucial to improve the knowledge and skills of the practitioners engaged in the relationship.

The first, process evaluation, involves the assessment of the mutual activity not only at the end but also during the activity. Findings uncovered that this process evaluation was needed to make the right improvements and changes, especially when the activity did not roll out according to plan. *“It can also be, which is currently the case for ‘integration runs’, that it doesn’t go as initially planned, and that a lot of drop out occurs. Then we sit together, to discuss what happened and how we can prevent this drop out from happening in the future.”* (PC1, CSP 1).

Trust was the second key element uncovered in the interview data. It refers to the mutual confidence in the abilities and intentions of partners. Findings indicated that higher trust led to more knowledge and skill sharing among the partners. Moreover, the analysis highlighted the influence of four other key elements to foster trust among the partners namely period of collaboration-time, personal contact, coordination and external focus. Period of collaboration-time is the first key element to foster trust, and refers to the period of time that partners have been collaborating to reach a common target. Many partners indicated that before sharing information a certain level of trust needed to be established. In most cases interviewees expressed that it took time to develop trust. *“In the beginning the youth non-profit organisations refused to invite me for their meetings… It was only after a few years, because I got to know and get along with several of the partners, that this perception changed and that I was invited to their meeting.” (City, CSP1)*

Personal contact, the second key element identified as central to fostering trust, relates to the personal relationships between the representatives of the CSP and representatives of the other organisations. Interviews uncovered that having a good personal ‘connection’ is needed to foster trust and to engage in mutual projects and share expertise. One organisation stated: *“… you need to have an informal connection to make the formal work… More often I have the impression that the match between people is more important than the content of the project they work on.”* (City, CSP 2). Personal attributes that were often mentioned as being highly valuable to make the partnership work were having an open attitude and being engaged in the relationship itself.

Coordination emerged as a third important element to foster trust among the practitioners. It refers to the clarity of the role, task and expected input in the relationship. Partners declared that they knew what was expected from them, and what benefit they received, which differed from other partnerships in which they were involved. “*One of the reasons why the collaboration is an added value is because it is concrete and clear, always tangible. Partnerships with other organisations often are somewhat cloudy and it is often difficult to see the organisation’s true intentions.”* (PC 2, SO2).

External focus was the fourth key element to foster trust. It covers the engagement of individuals in activities with partners to reach the goals of their own organisation. Our analysis revealed that people who were able to take a step back from their daily tasks and consider how they could represent added value for their partners multiplied trust and willingness to share knowledge, skills and information with that partner. *“The thing that really allowed people to know and trust person X was because person X frequented the places where our target group gathered. He further helped with the food distribution for the poor and he came to all our different meetings. When he told us that the best way for our target group to work with sport is to play netball, we followed his advice and we still play it today.” (PC 1, SO 2).*

### Key elements of cross-sector partnerships that build capacity at the organisational level

Two items were deduced from the analysis to build capacity at the organisational level: mutuality and policy support. These elements were found key to increase support and infrastructure.

The first, mutuality, describes the interdependence of the network partners. The analysis highlighted that the larger the interdependence and the perceived need to collaborate between the partners, the larger the willingness to share human, financial and infrastructural resources. *“Over the years we have put more emphasis on [civic, cultural, sports] participation. As a result, we received more [sport] questions from our clients, which put a heavy strain on our organisation. To cope with this problem we asked the CSP if one of their staff members could be incorporated in our organisation.”* (City, SO 2)

The second element, policy support, refers to the amount of resources that were allocated to the CSP by the policy makers. Interviews pointed out that support of the policy was in turn influenced by the support of partners and by metrics of success that could be presented to the policy makers. Policy directs a substantial part of the funding of public organisations and consequenty the sustainability and legitimacy of the partnerships and the CSP. As a result of a new policy agreement, the CSP was able to expand their work span from three to ten communities. “*I think the most important leap that we took was in 2007 with the new policy agreement… If the politicians chose not to invest in the CSP, then I don’t think that we would have had the basis to carry out such a wide program.” (City, CSP 3).*

Linked with the policy support is the support that partners give to the CSP: *“…but we have also grown because partners indicated that the CSP is a useful program which needs to be continued and financed. Policy and partners are very important to legitimize your existence.” (City, CSP 3).* A second item important to influence policy support were the objective results that could be presented to the policy makers. *“The city government did not cut the budgets of the CSP. This is in large part due to the fact they are able to present clear, objective results.” (City, SO 2).*

### Key elements of cross-sector partnerships that build capacity at the partnership level

Three elements found to build capacity at the partnership level were diversity of activities, partner complementarity and fit, and period of collaboration time. These elements were found important to increase the density and sustainability of the network.

A first key element uncovered in the interview data was the diversity of activities. It entailed the different activities that the CSP had to offer, which created added value for different partners in the different sectors. This ultimately led to attracting higher number of partners to the network. A ‘bike school’ (a course for adults to learn how to ride a bike), for instance, was particularly interesting for social centres who focused on empowering socially deprived groups, because it improved mobility of these people – an important element in employment. The sporting activities that the CSP organized together with youth organisations serving disadvantaged children, offered these children a structured leisure activity to which they were welcomed and that kept them off the streets. A ‘personal guidance activity’ benefited multiple health and social organisations by consulting, supporting and connecting their target group to the sport offered in their community, where they could participate and create social ties. Moreover, this activity aided sport organisations by helping them recruit new club members and developing the skill to deal with them appropriately.

A second key element identified by the analysis was the complementarity and fit between partners. This related to the composition of network partners and the harmonization between them. Findings suggest that the non-profit sport organisations on the one hand and the public youth, culture, health, social organisations on the other hand have many complementary skills to share. However interviewees indicated that before the CSP was implemented in the community these two types of organisations did not fit, mostly because the sports organisations did not have affinity with the disadvantaged target group. The CSP bridged this gap by sharing information from the youth, social, health partners to the sport organisations on how to deal with the disadvantaged target group, i.e., information on which thresholds they experience, which sporting needs they encounter, or how best to reach them. *“The added value [of the collaboration with the CSP] is the feedback the CSP gives. They have experience in dealing with projects with disadvantaged children and they give advice on problems we encounter” (PC 4, SP1).* Otherwise the CSP shared knowledge and skills from the sport organisations to the public organisations on bringing a customized sport program and information adjusted to the needs of their target group with respect for their thresholds to engage in sport participation. *“They know a wide variety of sports that we can’t offer with our background. The way they guide the activities always happens very professionally and is popular in the community.” (PC 1, CU1).*

The third element, period of collaboration-time, has earlier been described in building capacity at the practitioner level. However findings revealed that period of collaboration-time was also an important element to build capacity on the partnership level. Most partners expressed a growing interest and belief in the CSP as the relationship matured. This enhanced the legitimacy of the CSP and in turn attracted other organisations to work together with the CSP. *‘What helped is that people started to realise that the methods of the CSP deliver success. It takes time, because it is a totally different way of approaching people. For example if we organize sports camps you can’t participate if you haven’t paid. Contrarily the CSP will advance the payment, and sets up a payment plan for the ones that cannot pay’ (City, Sp 2).*

## Discussion

The main aim of this study was to explain how capacity was built through cross-sector partnerships. So far several studies have pointed out the effectiveness of capacity building in tackling health inequalities and sport promotion using cross-sector partnerships. However the specifics on how these partnerships build capacity is lacking. Therefore the present study researched the key elements of cross-sector partnerships that build capacity at the practitioner, organisational and partnership levels in a successful community sport program (CSP) that makes use of these partnerships.

At the practitioner level cross-sector partnerships have the potential to build capacity by sharing skills, knowledge and expertise among the partners inthe different sectors [14]. Our findings indicated that to build capacity at the practitioner level, process evaluation and trust are needed. We found process evaluation positively influencing skills and knowledge sharing among practitioners. Likewise, previous studies showed the importance of process evaluation to enhance organisational learning and capacity building [31]. Trust between partners was found to be an essential prerequisite to share knowledge and expertise. Throughout the literature different types of trust are described. The trust referred to in this study is relational trust. Bryk and Schneider [32] explain that engaging in relationships is engaging in dependencies and creating vulnerability for the individuals in the organization. Every deliberate action that reduces this sense of vulnerability fosters trust by making the individual safe and secure in their interactions [32]. Developing trust has been described as absolutely imperative to capacity building and one of the main principles of effective capacity building practice [33]. Additionally we found that trust was developed by a longer period of collaboration-time, a clear coordination, good personal contact, and an external focus.

Period of collaboration time is in Parent and Harvey’s [26] framework a subcategory of type of partnership. Many typologies exist in the partnership literature, among them the lifecycle of the partnership is central in understanding collaborative interactions [34]. Several studies indicate that in order to produce tangible results partnerships need time [22]. This study accentuated the importance of period of collaboration time in order to foster trust. In other studies period of time of collaboration is linked with sustainability of the collaboration, which is frequently used as a proxy for network effectiveness and a means for sustained health promotion effects [35–38]. Our results confirm the importance of period of collaboration time for capacity building. However, our results suggest that it should not be seen as an end, but as a potential catalyst to boost trust among practitioners and as a prerequisite to create legitimacy at the partnership level.

Coordination has been related to the set of tasks each party expects the other to perform [39]. Consistent with previous literature, we found that a clear role and task delineation resulted in higher trust to reach the mutual objectives of the partnership [39]. The interviews revealed personal contact as a third key element to foster trust.

Personal contact is an aspect of ‘staffing’ [26]. Parent and Harvey [26] specify that excellent staff support in the management of a partnership is critical to its success. Our analysis demonstrated that having a good personal ‘connection’ is needed to foster trust and to engage in mutual projects and share expertise. In particular, the match between individuals was found to e important. Foster-Fishman [40] earlier concluded that members with an open attitude and who were more committed to the partnership shared more information and skills. In our study these personality traits were found to enhance the match between the partners.

External focus has to our knowledge not been recognised as an important element of cross-sector partnerships to build capacity at the practitioner level. It goes beyond the initial contract of two partners working together to reach their own objectives through a partnership. Interviews revealed that an externally focused person is constantly looking for opportunities in his/her environment to create added value for his/her partners, but still initiating from his/her own expertise. S/He is flexible and an innovative champion with a shared problem orientation [41], taking collaborations to the next level. In other studies the importance of these community champions or change agents has also emerged [15, 42]. The value of neutral, credible, and legitimate intermediary leaders and intermediary organizations can create a collective impact and build capacity that multiplies health gains many times over [35, 43].

At the organisational level, mutuality and support of policy were found to be key elements of cross-sector partnerships to expand support and infrastructure. Interviews uncovered that social, health and other public organisations depended on the CSP to share resources concerning sport and vice versa. Babiak [44] earlier concluded that a higher interdependence between organisations results in more sharing of resources.

Support of politicians and policy is also recognized by other research as an important element to build capacity [45]. As suggested by Parent & Harvey [26], policy support is part of the ‘environment’ of a partnership. Environment is interpreted by these researcher as the political, demographic, economic, socio-cultural, legal, ecological and technological settings in which the collaboration operates. Our findings suggest that in particular, the political dimension had an influence on the amount of resources which were dispersed to the community sport program. Not in the least because policy makers fund these organisations [45]. According to our results support of politicians is closely linked to legitimacy of the partnership which is stimulated by the support of partners and metrics of success. These factors have proven their relevance for building capacity in other studies [37, 40].

At the partnership level, the main capacity builders of cross-sector collaborations were partner complementarity and fit, diversity of activities, and period of collaboration-time. These elements were found to build and strengthen partnerships and cohesiveness among the different organisations [14].

Previous research showed that the challenge of community-based organisations resides in the fact that they need to fit the complementary skills and knowledge of different types of organisations in order to collaborate [46]. This is even more true in sport promotion, as additional cultural differences between public and non-profit sport organisations make it hard for them to fit and to interact [47]. The CSP however managed to bridge this cultural gap and was a conduit of information and knowledge sharing between sport and public partners, which made it possible for these organisations to collaborate.

According to Provan [48] and others [49] forming partnerships begins with creating organisational benefits for partners. Our findings suggest that the CSP created organisational benefits for a diversity of partners because they engaged in a wide variety of activities, each creating different value for the partners collaborating in the CSP. This is congruent with the idea of ‘enlightened self interest’, wherein the best way to promote one’s own interest is by advancing the interests of others, and vice versa [50]. In the case of the CSP more organizations became interested in joining or enforcing the partnership when self-interest and own organizational benefits could be attained.

As mentioned earlier, period of collaboration-time of the partnership was found to boost legitimacy. In turn legitimacy is one of the important motives for entering into a sports-based partnership. With reference to the CSP, the program needed time to be able to show results and create legitimacy. Once results were shown, more partners were willing to collaborate with the CSP, and with more critical mass, even more positive results could be acquired, This notion is closely linked to the phenomenon of the ‘Matthew effect', which indicates that advantage breeds more advantage [51]. Parent and Harvey [26] earlier emphasized that formation, management and evaluation of partnerships are to be seen in a constant feedback loop. Labonte [52] affirms that in order for community capacity building initiatives to be successfully implemented and sustained, communities must possess or develop the capacity for collective action, the internal resources to support the process, and the necessary skills and knowledge to successfully identify local problems and their solutions. The idea that ‘it takes capacity to develop capacity’ is generally accepted in capacity building theory [53, 54].

The main limitation of this study is the issue of external validity and transferability of the findings. This study looked at a CSP in the specific context of disadvantaged communities. Other studies of other programs tackling health inequalities in other settings are needed to confirm or contradict the robustness of our findings. Another restraint is the limited focus on the competences of the people interacting in the partnership. The outcome of partnerships ultimately rests on the shoulders of those doing the program implementation [55]. Competences (e.g., motivation, skills, expertise, …) of the representatives of each organization are known to influence the overall partnership effectiveness [56]. Although we did differentiate which key elements of partnerships build skills and knowledge at the practitioner level, a more in depth understanding would probably be gained by researching how the competences of the people in the partnership influence the built capacity. Future studies are encouraged to elaborate on how and which competences are key for to build capacity at the different levels.

## Conclusions

Our study contributes to theory by giving insights into how capacity can be built on different levels through cross-sector partnerships in sport promotion. To the best of our knowledge, this distinction of how capacity can be built through cross-sector partnerships at the practitioner, organisational and partnership level has not been studied in previous research. This study further differentiates from other work done in this area by including perspectives from multiple partners and different stages of development in a sports promotion context.

Our findings contribute to practice by suggesting several actions which might be taken by organisations that aim to build capacity at different levels. First, at the practitioner level more knowledge is gained between organisations who evaluate their mutual activities during the process, and who foster mutual trust by having an open attitude towards the partners. Additionally, capacity is fostered when organizations have clarity about their role in the partnership, looki for opportunities in the environment and understand that trusting relationship takes time to be built. Second, at the organisational level, partners need to create interdependence between each other and build support from policy by getting support from other partners and having objective metrics that prove their value. Third, at the partnership level, organisations need to fit their complementary skills, diversify their activities and create credibility by delivering added value which takes time to be created. Specific for the context of sport promotion it is crucial to have an organisation that acts as a conduit of knowledge to bridge cultural differences between sports organisations on the one hand and health, social, culture and youth organisations on the other.

## Abbreviations

CSP: Community sport program
SP: Sport organisation
SO: Social organisation
CU: Cultural organisation
HE: Health organisation
YO: Youth organisation
PC: Program community
